# Study on the mechanism of mining-induced pressure mitigation in gangue backfill mining and the sensitivity analysis of main controlling factors

**DOI:** 10.1371/journal.pone.0327157

**Published:** 2025-07-11

**Authors:** Lei Zhu, Yunong Xu, Chengyong Liu, Yuejin Zhou, Wenzhe Gu, Cunli Zhu, Zhicheng Liu

**Affiliations:** 1 China Coal Technology and Engineering Group Xi’an Research Institute Co., Ltd, Xi’an, China; 2 State Key Laboratory for Geomechanics & Deep Underground Engineering, Xuzhou, China University of Mining & Technology, Xuzhou, China; 3 School of Mines, Xuzhou, China University of Mining & Technology, China University of Mining & Technology, Xuzhou, China; Shenyang Jianzhu University, CHINA

## Abstract

Gangue backfill mining technology represents a significant advancement in green mining, mitigating mine pressure and rock mass movements, thereby ensuring underground production safety. To investigate the mechanism of pressure reduction and the sensitivity of factors in gangue backfill mining, a mechanical model was first developed. Then, a method for calculating mining pressure in gangue backfill mining was derived based on the stress characteristics. Numerical simulation methods were then employed to analyze the patterns of mining pressure reduction. Finally, sensitivity analysis was performed using range analysis and variance analysis to determine the sensitivity of factors. The results indicate that: (1) The manifestation of mining pressure in gangue backfill mining is influenced by factors such as mining height and backfill collapse ratio; (2) Under the support of coal gangue, the concentrated stress in the coal seam significantly decreases, forming an arched shape according to the mining stages; (3) The range of plastic failure in the coal seam remains relatively stable under gangue backfill mining, with the plastic zone of the roof plate exhibiting a strip-like distribution; (4) Both range analysis and variance analysis revealed that the sensitivity ranking is backfill collapse ratio > mining height > elastic modulus. Variance analysis further confirms that mining height and backfill collapse ratio have significant impacts, while the elastic modulus of coal gangue has a negligible impact. The study analyzed the manifestation law of coal seam pressure under backfill mining and revealed the sensitivity of the main control factors, which can provide theoretical support for the stability control of coal seams under backfill mining.

## 1. Introduction

Under the influence of mining activities, the overlying rock strata above a coal seam undergo varying degrees of compression and deformation, leading to the formation of fissures and even collapses. These phenomena can readily cause significant deformation of the coal seam roof and floor, impact damage and other hazardous accidents, posing a threat to mine safety [1–5]. The application of gangue backfill mining technology not only attenuates the manifestation of mining-induced pressure but also indirectly protects the surface environment and reduces economic losses, aligning with sustainable development strategies [6–11]. However, despite this technology, the internal stress balance can still be disrupted, resulting in a redistribution of stresses in the overlying rocks and leading to stress concentration. This stress concentration manifests directly as periodic weighting and basic roof collapses [12–14]. Therefore, studying the pressure manifestation in gangue backfill mining is pivotal for understanding the movement patterns of rock masses under mining-induced effects. To ensure the safety of coal mine production, there is an urgent need to investigate the weakening mechanisms of mining-induced pressure and the sensitivity of the main controlling factors.

In the 1960s, countries such as Germany and Poland were the first to adopt backfill technology, significantly reducing mining damage [15–18]. China began systematic research on backfill mining in the 1980s and successfully applied it in mining areas such as Xinwen and Yanzhou [19,20]. With the advancement of technology, the filling method has evolved from lagging behind in the early goaf filling to simultaneous filling during mining. Weakening mining pressure is one of the core advantages of gangue filling mining, which supports the overlying rock through the filling body, reduces the strength of mining pressure manifestation on the working face [18,21]. The cyclic compression step distance and strength of the gangue filling working face are significantly smaller than those of the collapse mining method, and the stability of the roof is significantly improved [22,23].

In order to reveal the weakening law of mining pressure under backfill mining, scholars have conducted research at different levels through theoretical analysis, numerical simulation, and on-site monitoring methods, and have achieved rich results in controlling rock strata in backfill mining. Miao Xiexing et al. [24] introduced the “equivalent mining height” theory, conceptualizing the entire process of backfilled coal mining as the extraction of an extremely thin coal seam working face, thereby innovatively representing the equivalent concept in solid-filled coal mining. Zhang Qiang et al. [25–27] defined the concept of critical filling ratio for backfilled mining areas, with monitoring results validating the feasibility of the theory. Xu Jialin et al. [28,29] proposed partial filling mining technology, which reduces the use of filling materials through rational layout, thereby lowering filling costs and achieving coal resource recovery, economic efficiency, and mine safety. Zhang Jixiong et al. [30] systematically addressed theoretical issues related to green mining of deep coal resources and established a technical framework for gangue backfill mining. Qi Hegang et al. [31] explored the “short filling, long mining” method, effectively addressing the waste of residual coal pillars and the accumulation of gangue on the surface, thereby establishing a new model for green mining. Yang Shengli et al. [32], using the 1123 backfilled working face of the Gucheng Coal Mine as a case study, employed theoretical analysis, laboratory experiments, and field measurements to investigate the impact of filling support on the movement patterns of hard roofs and explored control methods for low-lying hard roofs. Tang Yuesong et al. [33], aiming to reduce the impact of rock bursts on the safe mining of burst-prone coal seams, conducted field measurements, theoretical analysis, and laboratory experiments to study the mechanisms of sudden stress increases and drops under deep backfilled mining conditions, exploring the synergistic pressure relief effects of pre-splitting blasting and large-diameter drilling. Yang Ke et al. [34], using a similar material simulation experiment and theoretical analysis, studied the temporal and spatial evolution laws and mechanical mechanisms of stress fields, displacement fields, and fracture fields in the overlying rock during backfilled mining at a test working face in a mine in Ningxia.

These studies reveal the evolution of stress fields and the movement patterns of overlying strata in backfill mining. However, in the design of working conditions, there is a lack of research on the weakening mechanism of mining pressure and the sensitivity of influencing factors under the influence of multiple factors, especially the main controlling factors that play a major role in the manifestation of mining pressure. To analyze the mechanism of pressure mitigation in gangue backfill mining, a computational mechanical model was first established, grounded on the concept of advanced abutment pressure during mining activities. From this model, a methodology for calculating mining-induced pressure in backfill mining was derived. Subsequently, numerical simulations were conducted, leveraging the specific stratigraphic conditions of a coal mine in Pingmei mining area, to analyze the patterns of pressure mitigation in gangue backfill mining. Lastly, orthogonal experiments were designed targeting the main controlling factors. Through a comparative analysis employing range analysis and variance analysis, the sensitivity of each Main controlling factor to the manifestation of mining-induced pressure in backfill mining was determined. These results can provide theoretical support for the stability control of coal seams under backfill mining

## 2 Mechanism of mining-induced pressure manifestation in gangue backfill mining

### 2.1 Construction of computational mechanics model

**Fig 1:**
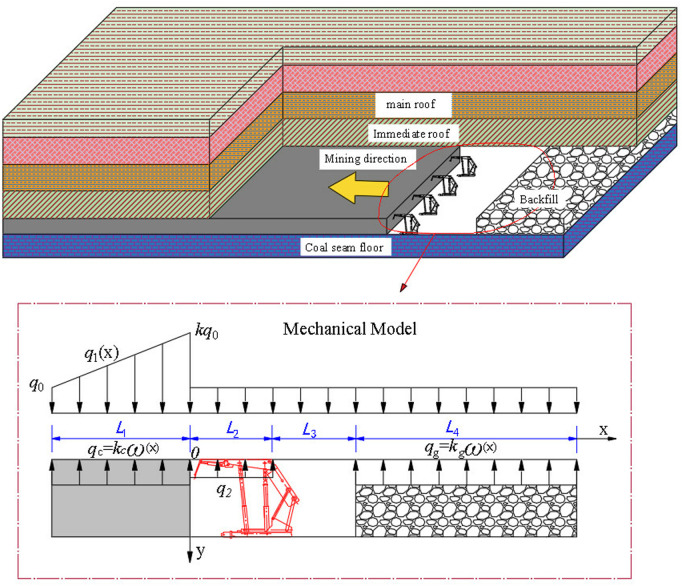
Mechanical model for studying strata pressure behavior in gangue backfill mining.

The research model for coal seams under backfill mining is much larger than that of heterogeneous rock units, so it is assumed that the rock layers are uniformly distributed. Due to the effects of mining-induced stress, an advanced abutment pressure zone forms both ahead of and behind the mining face [35]. Based on the distribution pattern of this abutment pressure, a mechanical model for mining-induced pressure manifestation under the conditions of backfill mining is established [36]. In this model, the origin of the coordinate system is positioned at the intersection of the advancing mining face and the coal seam floor. The X-axis represents the direction of mining face advancement, while the Y-axis points towards the deeper direction (Fig 1).

In Fig 1, *L*_1_ is the length of the non-elastic zone of the coal body, measured in meters, *L*_2_ is the length of the hydraulic support for filling and mining, measured in meters, *L*_3_ is the distance between the control roof, measured in meters, *L*_4_ is the length of the filling body in the goaf, measured in meters, *q*_0_ is the original rock stress of the overlying rock layer acting on the roof, measured in MPa, *q*_1_(*x*) is the advanced concentrated stress, measured in MPa, *q*_2_ is the support force of the hydraulic support for the roof, measured in MPa, *k* is the stress concentration factor, no unit, and ω(x) is the subsidence deflection of the roof in the filling mining area, measured in meters.

Under the influence of mining activities, an advanced concentrated stress $q_{1}(x)$ arises in the area ahead of the mining face.


$$q_{1}(x)=\frac{(k-1)q_{0}}{L_{1}}x+kq_{0}$$ (1)

Based on the interaction relationship between hydraulic support, coal body, and filling body in deep filling mining, the relationship between compression and resistance can be obtained by approximately replacing the filling body with a Winkler foundation [37]:


$$q_{c}=k_{c}\omega (x)$$ (2)

$$q_{g}=k_{g}\omega (x)$$ (3)

$k_{c}$ and $k_{g}$ denote the foundation coefficients for the coal seam and coal gangue, respectively. The formulas for calculating $k_{c}$ and $k_{g}$ are as follows:


$$k_{c}=\frac{E_{c}}{h}$$ (4)

$$k_{g}=\frac{E_{g}}{h_{g}}$$ (5)

where $E_{c}$ represents the elastic modulus of the coal pillar (GPa); $E_{g}$ represents the elastic modulus of the coal gangue (GPa); h denotes the mining height (in m); and $h_{g}$ denotes the height of the fill material (m).

### 2.2 Calculation method for strata pressure in gangue backfill mining

Under the action of the load *q*_0_ from the overlying rock strata, the pressure *q*(*x*) exists between the beam of the elastic foundation and the foundation. At this point, the subsidence deflection equation for the immediate roof can be expressed as:


$$EI\frac{d^{4}\omega (x)}{dx^{4}}=q_{0}-q(x)$$ (6)

In the equation, ω represents the subsidence deflection (m); E denotes the elastic modulus of the roof (GPa); and *I* represents the moment of inertia of the roof beam ($m^{4}$).

For the stage -*L*_1_−0:


$$EI\frac{d^{4}\omega (x)}{dx^{4}}+k_{c}\omega (x)=q_{1}(x)=\frac{(k-1)q_{0}}{L_{1}}x+kq_{0}$$ (7)

Let $\alpha =\sqrt[{4}]{\frac{k_{c}}{4EI}}$ be a characteristic coefficient, and solve the differential equation.


$$w_{1}(x)=e^{-\alpha x}[A_{1}\cos(\alpha x)+B_{1}\sin(\alpha x)]+e^{\alpha x}[C_{1}\cos(\alpha x)+D_{1}\sin(\alpha x)]+\;\frac{q_{1}(x)}{k_{c}}$$ (8)

*L*_1_ can be conceptualized as a semi-infinite beam. When a certain condition x→−∞ is met, the subsidence of the roof beam above the coal wall converges to a constant value, denoted as $A_{1}=B_{1}=0$. Under this condition, the deflection of the roof beam above the coal wall can be simplified as follows:


$$w_{1}(x)=e^{\alpha x}[C_{1}\cos(\alpha x)+D_{1}\sin(\alpha x)]+\;\frac{q_{1}(x)}{k_{c}}$$ (9)

For the stage 0-*L*_2_:


$$EI\frac{d^{4}\omega (x)}{dx^{4}}+q_{2}(x)=q_{0}$$ (10)

Solve the differential equation:


$$w_{2}(x)=\frac{(q_{0}-q_{2})x^{4}}{24EI}+A_{2}x^{3}+B_{2}x^{2}+C_{2}x+D_{2}$$ (11)

For the stage *L*_2_- *L*_2_ + *L*_3_:


$$EI\frac{d^{4}\omega (x)}{dx^{4}}=q_{0}$$ (12)

Solve the differential equation:


$$w_{3}(x)=\frac{q_{0}x^{4}}{24EI}+A_{3}x^{3}+B_{3}x^{2}+C_{3}x+D_{3}$$ (13)

For the stage *L*_2_ + *L*_3_- *L*_2_ + *L*_3_ +* L*_4_:


$$EI\frac{d^{4}\omega (x)}{dx^{4}}+k_{g}\omega (x)=q_{0}$$ (14)

Let $\beta =\sqrt[{4}]{\frac{k_{g}}{4EI}}$ be a characteristic coefficient, and solve the differential equation:


$$w_{4}(x)=e^{-\beta x}[A_{1}\cos(\beta x)+B_{1}\sin(\beta x)]+e^{\beta x}[C_{1}\cos(\beta x)+D_{1}\sin(\beta x)]+\;\frac{q_{0}}{k_{g}}$$ (15)

*L*_4_ can be considered as a semi-infinite beam. When x→∞, the subsidence of the roof beam above the coal wall tends towards a constant value. At this point, the deflection of the roof beam above the coal wall can be simplified as follows:


$$w_{4}(x)=e^{-\beta x}[A_{4}\cos(\beta x)+B_{4}\sin(\beta x)]+\;\frac{q_{0}}{k_{g}}$$ (16)

The relationships among the rotation angle *θ*, bending moment *M*, shear force *Q*, and deflection *ω* at any cross-section within the beam can be expressed as follows:


$$\theta =\frac{d\omega (x)}{dx}$$ (17)

$$M=-EI\frac{d^{2}\omega (x)}{dx^{2}}$$ (18)

$$Q=-EI\frac{d^{3}\omega (x)}{dx^{3}}$$ (19)

Considering the boundary conditions and continuity constraints among the various sections of the top beam, it can be deduced that:


$$\begin{array}{c} \left\{ \;\;\;\begin{array}{c} \omega_{1}(0)=\omega_{2}(0)=0,\theta_{1}(0)=\theta_{2}(0)=0,M_{1}(0)=M_{2}(0),Q_{1}(0)=Q_{2}(0),\mathrm{\backslash vspace}0.5mm\\ \mathrm{\backslash vspace}0.5mm\omega_{2}(L_{2})=\omega_{3}(L_{2}),\theta_{2}(L_{2})=\theta_{3}(L_{2}),M_{2}(L_{2})=M_{3}(L_{2}),Q_{2}(L_{2})=Q_{3}(L_{2}),\\ \mathrm{\backslash vspace}0.5mm\omega_{3}(L_{2}+L_{3})=\omega_{4}(L_{2}+L_{3}),\theta_{3}(L_{2}+L_{3})=\theta_{4}(L_{2}+L_{3}),\\ \mathrm{\backslash vspace}0.5mmM_{3}(L_{2}+L_{3})=M_{4}(L_{2}+L_{3}),Q_{3}(L_{2}+L_{3})=Q_{4}(L_{2}+L_{3}) \end{array}\right. \end{array}$$ (20)

For the stage -*L*_1_ ≤ x ≤ 0:


$$\begin{array}{c} \left\{ \;\;\;\begin{array}{c} w_{1}(x)=e^{\alpha x}[C_{1}\cos(\alpha x)+D_{1}\sin(\alpha x)]+\;\frac{q_{1}(x)}{k_{c}}\mathrm{\backslash vspace}5mm\\ \mathrm{\backslash vspace}5mm\theta_{1}(x)=e^{\alpha x}[(D_{1}-C_{1})\sin(\alpha x)+((C_{1}+D_{1})+\cos(\alpha x)]+\;\frac{(k-1)q_{0}}{L_{1}k_{c}}\\ M_{1}(x)=-2EI\alpha^{2}e^{\alpha x}[D_{1}\cos(\alpha x)+C_{1}\sin(\alpha x)]\\ Q_{1}(x)=-2EI\alpha^{3}e^{\alpha x}[(D_{1}-C_{1})\cos(\alpha x)+(D_{1}+C_{1})\sin(\alpha x)] \end{array}\right. \end{array}$$ (21)

Based on $\omega_{1}(0)=0$, $C_{1}=-\frac{kq_{0}}{k_{c}}$ can be derived; similarly, from $\theta_{1}(0)=0$, $\alpha (D_{1}+C_{1})+\frac{(k-1)q_{0}}{k_{c}L_{1}}=0$ can be obtained. Combining the above equation, the following can be obtained:


$$D_{1}=\frac{(k\alpha L_{1}-k+1)q_{0}}{\alpha k_{c}L_{1}}$$ (22)

Based on this method, the eigenvalue expressions for the stages of 0-*L*_2_, *L*_2_-*L*_2_ + *L*_3_, and *L*_2_ + *L*_3_-*L*_2_ + *L*_3_ + *L*_4_ are calculated sequentially. Subsequently, the deflection and stress magnitudes at various points along the beam are derived.

## 3 Weakening law of mining pressure in gangue backfill mining

### 3.1 Establishment of numerical model for gangue backfill mining

Based on FLAC^3D^, a numerical simulation model is established, and the model is set to 200m × 500m × 100m considering the influence of boundary effects. To fully understand the stress distribution and failure characteristics at the top and bottom of the coal seam, refine the grid at the top and bottom of the coal seam, dividing two grids per meter and one grid per meter in other areas. The full mining and filling method is adopted for the advancement of the working face.

**Fig 2:**
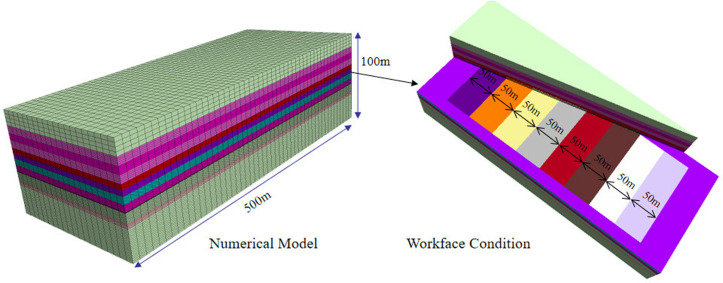
Establishment of Numerical Model.

According to the actual condition of Pingmei coal seam, 50m coal pillars are left on both sides of the coal seam, with a pushing distance of 400m. The model is fixed in the x and y directions in the left, right, front, and rear directions, and the bottom is a fully constrained boundary; To improve the computational resolution of large-scale models, the actual existence of heterogeneous rock layers such as joints, fractures, and bedding is simplified into a uniform continuous medium; Deep mining is a major component of the stress field, and the influence of tectonic stress is relatively small. Therefore, the original stress field only considers the self weight stress field and does not take into account the tectonic stress field; The coal seam is deeply buried and dominated by self weight stress. It can be reasonably assumed that the lateral pressure coefficient is 1(Fig 2).

Assuming that the surrounding rock is of ideal elastic-plastic material, the Mohr-Coulomb yielding criteria are adopted:


$${\sigma_{1}}^{\prime }-{\sigma_{3}}^{\prime }=2c\cot\varphi -({\sigma_{1}}^{\prime }+{\sigma_{3}}^{\prime })\sin\varphi$$ (23)

Where $\sigma_{1}^{\prime }$ represents the maximum principal stress obtained from numerical calculations (MPa); $\sigma_{3}^{\prime }$ represents the minimum principal stress obtained from numerical calculations (MPa); c represents the cohesive force of the rock (MPa); *φ* represents the internal friction angle of the rock.

Using a coal seam in the Pingmei mining area as the research context, the basic mechanical parameters for this area were adopted. Based on th e conditions of the rock strata forming the roof and floor of the coal seam, the mechanical parameters utilized are presented in Table 1.

**Table 1:** Basic mechanical parameters of rocks.

Lithology	Bulk Modulus/GPa	Shear Modulus/GPa	Cohesion/MPa	Internal Friction Angle/(°)	Tensile Strength/MPa	Bulk DensityN/m³
Limestone	5.68	4.24	9.2	34	7.1	2750
Sandstone	2.89	2.54	3.2	40.9	3.7	2977
Limestone	5.68	4.24	9.2	34	7.1	2750
Mudstone	9.97	7.35	1.2	32	0.58	2483
Coal Seam	4.91	2.01	1.25	32	0.15	1380
Mudstone	9.97	7.35	1.2	32	0.58	2483
Limestone	5.68	4.24	9.2	34	7.1	2750
Mudstone	9.97	7.35	1.2	32	0.58	2483
Fine Sandstone	21	13.52	3.2	40	1.29	2580

Gangue is selected as the backfill material, the basic mechanical parameters is shown in Table 2.

**Table 2:** Basic parameters of backfill material.

Lithology	Bulk Modulus /GPa	Shear Modulus/GPa	Cohesion/MPa	Internal Friction Angle/(°)	Tensile Strength/MPa	Bulk Density N/m³
Gangue	1.0	5.56	5.8	34	5.1	2550

### 3.2 Weakening law of stress field for gangue backfill mining

After coal seam extraction, three zones—the caving zone, fracture zone, and bending-subsidence zone—sequentially form vertically from the bottom up along the goaf, dynamically advancing with the progression of the longwall working face. This process constitutes a continuous evolution. To analyze the weakening mechanism of stress concentration in the coal seam as a result of gangue backfilling, a comparison was made between the stress evolution in the coal seam before and after filling.

**Fig 3:**
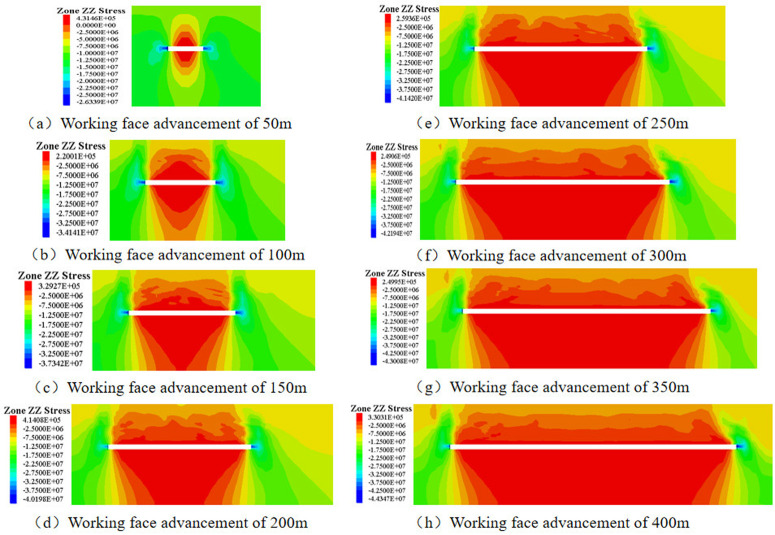
Stress Evolution in the Coal Seam Without Gangue Backfill.

The stress evolution pattern of the coal seam, without the influence of gangue backfill (Fig 3), which depicts vertical slices taken at *x* = 0. Prior to coal extraction, the overburden rock layers maintained a state of stress equilibrium extending to the surface. As the longwall working face progresses, the stress within the rock-soil mass undergoes redistribution, resulting in alterations to the stress state of the coal seam’s roof and floor. Notably, stress concentration emerges at the center of the goaf, oriented vertically upwards, and intensifies with increasing advance distance. Specifically, vertical stress concentration peaks on both sides of the coal pillar, with the concentrated stress rising from 21.53 MPa at an advance distance of 50m to 42.3 MPa at 400m.

**Fig 4:**
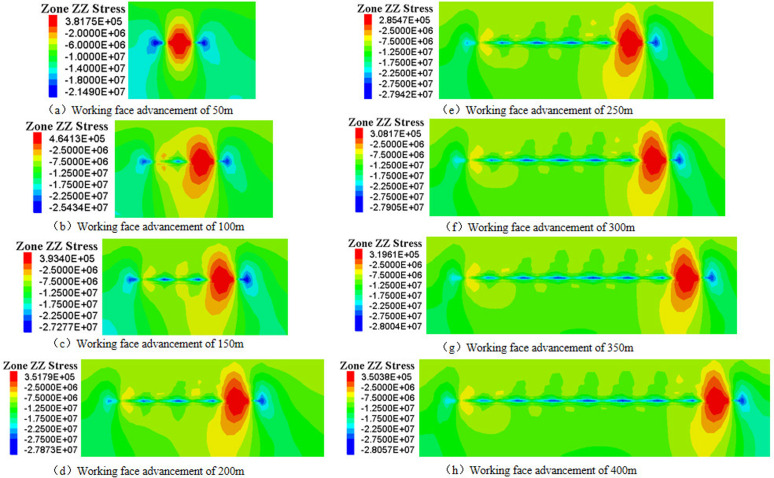
Stress evolution in coal seam under the influence of gangue backfill.

The stress evolution in the coal seam under the influence of gangue backfill is shown (Fig 4). With the support provided by the gangue backfill, the vertical stress within the coal seam experiences a notable reduction compared to conventional caving mining methods. The stress distribution pattern exhibits an arched shape at each construction stage within the model. At an advance distance of 50 meters, the maximum vertical stress is concentrated on either side of the coal pillar, approximately 22.5 MPa. Stress concentration also occurs in the center of the backfilled area, with the floor experiencing a greater degree of disturbance compared to the roof. Upon advancing to 100 meters, the stress in the roof and floor of the backfilled section decreases significantly. Stress concentration is observed at the critical points of the fill, reaching approximately 26.3 MPa, while the stress ahead of the working face peaks at 27.5 MPa. As the mining operation progresses further, the stress distribution pattern in the roof and floor remains largely unchanged. The maximum vertical stress increases gradually, from 27.5 MPa at 100 meters to 30.0 MPa at 400 meters. When compared to caving mining, the maximum vertical stress in the coal seam after gangue backfill is approximately 70.91% of that observed in caving mining conditions. Stress concentration areas are prone to rockburst, so in practical engineering, advance support should be provided at the coal seam mining line and working face to prevent rockburst accidents.

### 3.3 Weakening law of plastic zone for gangue backfill mining

Under backfill mining conditions, gangue can serve as a substitute for the original coal seam, providing essential support stress to the overlying rock layers. This support effectively minimizes the degree of deformation within the overlying rock layers, which in turn reduces the extent of surface deformation and damage. To analyze the weakening mechanism of gangue backfill on overlying rock damage, a comparison was made between the plastic zones of the coal seam before and after the introduction of gangue backfill.

**Fig 5:**
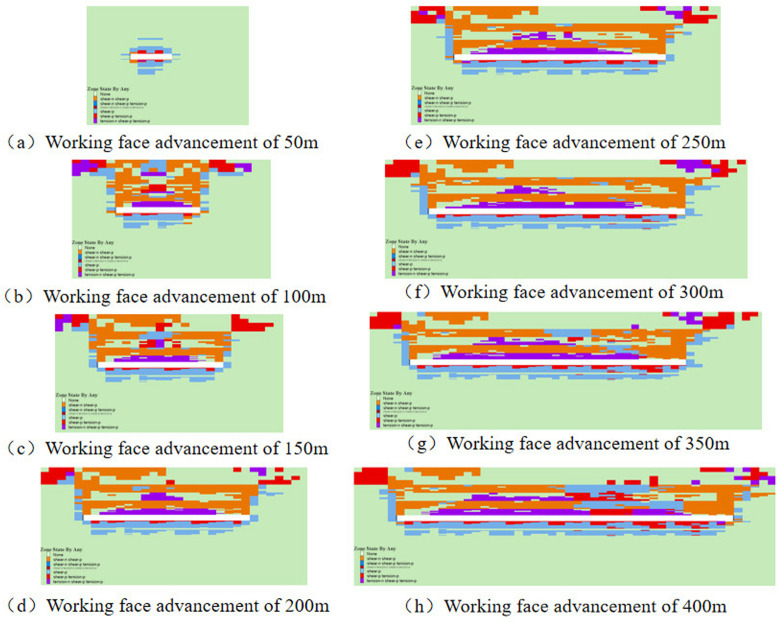
Distribution of plastic zones in coal seam without gangue backfill.

The distribution of plastic zones and stress in the coal seam without gangue backfill is shown (Fig 5). Under the influence of mining, the basic roof undergoes significant bending deformation or fracture, with the plastic zone primarily exhibiting tensile-shear failure, while overall failure remains predominantly tensile. Moreover, The floor mainly exhibits shear failure affected by coal seam mining. As the advance distance increases, the depth of roof and floor damage increases. At an advancement of 50m, the damage depth of the roof and floor is relatively uniform and minimal, with plastic zone depths approximately measuring 3.2m and 1.6m, respectively. Upon reaching an advancement of 100m, due to the roof composition of relatively low-strength soft rock, a through-going plastic zone forms, while the floor damage depth reaches approximately 15.7m. Throughout the advancement process, the height of roof damage progressively increases, whereas the depth of floor damage initially rises and then stabilizes around 100m.

**Fig 6:**
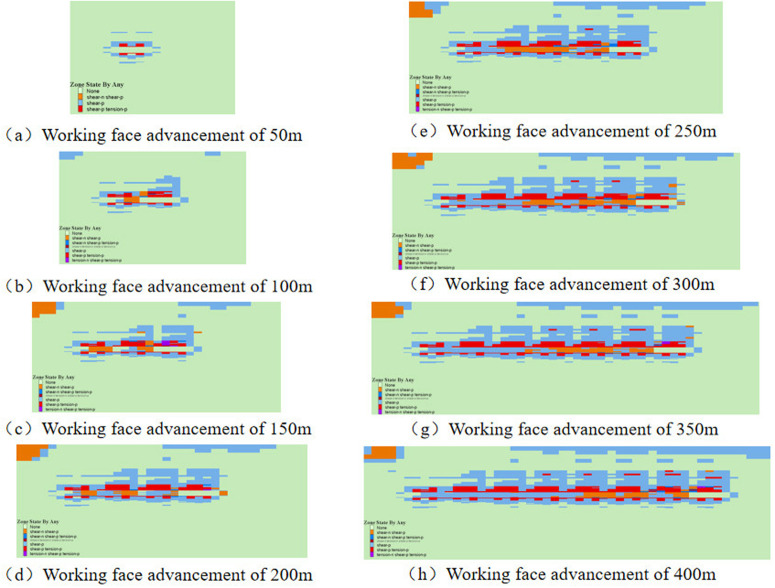
Distribution of plastic zones in coal seam with gangue backfill.

The distribution of plastic zones within a coal seam subjected to gangue backfill mining is shown (Fig 6). When the mining face advances by 50m, the extent of the plastic zone in the coal seam is comparable to that observed in non-backfilled conditions. Upon advancing to 100m, the roof experiences fracturing, resulting in a plastic zone depth of approximately 16.4m, while the plastic damage depth of the floor remains relatively stable at around 5.7m. As the mining face continues to advance further, the depth of damage to both the roof and floor remains virtually unchanged, with the roof maintaining a consistent depth of 16.4m and the floor at 5.7m. The plastic zones in the roof and floor of the coal seam exhibit an elongated shape and are predominantly characterized by shear failures, without the presence of large-scale arched shaped damage. Compared to conventional caving mining methods, the extent of the plastic zones is significantly reduced under gangue backfill conditions. This observation highlights the stabilizing effect of gangue backfill on the coal seam, mitigating the extent of plastic deformation and damage in the surrounding rock mass. When a plastic zone appears in the rock layer, it means that damage has been caused. Therefore, in practical engineering, it is necessary to strengthen the support at the top and bottom plates after filling and mining to ensure the stability of the coal seam. The conclusions drawn by previous researchers based on similar simulations and field experiments are similar to those in this article [38,39].

## 4 Sensitivity analysis of the main controlling factors of mining pressure manifestation in orthogonal gangue backfill mining

### 4.1 Orthogonal experimental design

The primary factors influencing the manifestation of mining stress in fill mining include mining parameters and filling technology. Mining dimensions such as mining width, length and height are considered, while filling technology includes the properties of filling materials and techniques.

Based on the mechanical calculation model shown in Fig 1, the backfill collapse ratio is denoted as:


$$\eta =\frac{L_{4}}{L_{2}+L_{3}}$$ (24)

Based on Equation (21), the main controlling factors chosen are mining height, backfill collapse ratio, and the elastic modulus of the filling material. The specific level values selected for each of these main controlling factors are presented in Table 3.

**Table 3:** Levels of main controlling factors influencing stress manifestation.

Main Control Factor	Level 1	Level 2	Level 3
*A*, Mining Height/m	*A*_*1*_ (3)	*A*_*2*_ (5)	*A*_*3*_ (7)
*B*, Backfill Collapse Ratioη	*B*_*1*_ (60%)	*B*_*2*_ (80%)	*B*_*3*_ (100%)
*C*, Elastic Modulus of Waste Rock/GPa	*C*_*1*_ (0.5)	*C*_*2*_ (1)	*C*_*3*_ (1.5)

For multi-factor and multi-level experimental requirements, to analyze the significance and influence patterns of various factors on the evaluation indicators, previous researchers have often employed orthogonal testing for efficient and rapid analysis [40–42]. In addition to mining height, backfill collapse ratio and elastic modulus of the filling material, an extra column is reserved as an empty column for the purpose of analyzing experimental errors. Thus, an orthogonal table with four factors and three levels is established. The orthogonal experimental design scheme is shown in Table 4.

**Table 4:** Orthogonal experimental design for pressure manifestation analysis.

Experiment Group	Main Control Factor Levels			
	Mining Height	Backfill Collapse Ratio	Elastic Modulus of Filling Body	Empty Column
1	*A*_*1*_ (3m)	*B*_*1*_ (60%)	*C*_*1*_ (0.5GPa)	1
2	*A*_*1*_ (3m)	*B*_*2*_ (80%)	*C*_*3*_ (1.5GPa)	2
3	*A*_*1*_ (3m)	*B*_*3*_ (100%)	*C*_*2*_ (1.0GPa)	3
4	*A*_*2*_ (5m)	*B*_*1*_ (60%)	*C*_*3*_ (1.5GPa)	3
5	*A*_*2*_ (5m)	*B*_*2*_ (80%)	*C*_*2*_ (1.0GPa)	1
6	*A*_*2*_ (5m)	*B*_*3*_ (100%)	*C*_*1*_ (0.5GPa)	2
7	*A*_*3*_ (7m)	*B*_*1*_ (60%)	*C*_*2*_ (1.0GPa)	2
8	*A*_*3*_ (7m)	*B*_*2*_ (80%)	*C*_*1*_ (0.5GPa)	3
9	*A*_*3*_ (7m)	*B*_*3*_ (100%)	*C*_*3*_ (1.5GPa)	1

Support stress peak value indicates the state of stress concentration, and taking the support stress peak value as the comparison standard can reflect the state of mine pressure under the influence of mining.

**Fig 7:**
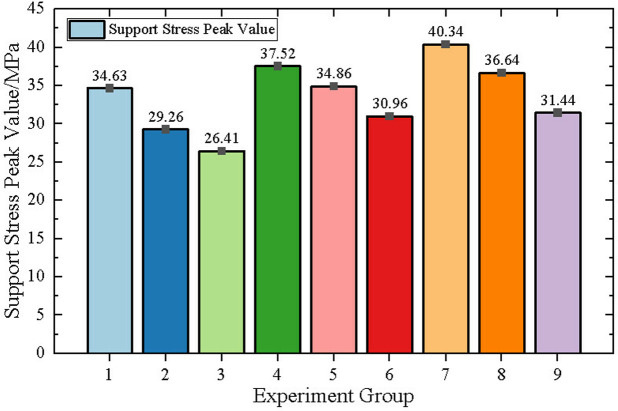
Orthogonal test results diagram.

In order to clearly analyze the peak stress values of different experimental groups, the experimental results are shown in Fig 7. group 3 has the smallest mine pressure and the support stress peak value is 26.41MPa, and group 7 has the largest mine pressure and the support stress peak value is 40.34MPa.

Range analysis and variance analysis are the most commonly data processing methods for orthogonal experiments. Both methods can assess the sensitivity of the main controlling factors. To ensure the accuracy of the sensitivity study, this paper employs both range analysis and variance analysis for comparative research.

### 4.2 Range analysis of main controlling factors influencing pressure manifestation in gangue backfill mining

Range analysis shows the maximum potential variation in data, and aids in a quick understanding of data fluctuations [31]. The range analysis for the main controlling factors influencing stress manifestation in gangue backfill mining is shown in Table 5.

**Table 5:** Range Analysis of Main Controlling Factors in Pressure Manifestation.

Experiment Group	Main Control Factor Levels				
	Mining Height	Backfill Collapse Ratio	Elastic Modulus of Filling Body	Empty Column	Support Stress Peak Value/MPa
1	*A*_*1*_ (3m)	*B*_*1*_ (60%)	*C*_*1*_ (0.5GPa)	1	34.63
2	*A*_*1*_ (3m)	*B*_*2*_ (80%)	*C*_*3*_ (1.5GPa)	2	29.26
3	*A*_*1*_ (3m)	*B*_*3*_ (100%)	*C*_*2*_ (1.0GPa)	3	26.41
4	*A*_*2*_(5m)	*B*_*1*_ (60%)	*C*_*3*_ (1.5GPa)	3	37.52
5	*A*_*2*_ (5m)	*B*_*2*_ (80%)	*C*_*2*_ (1.0GPa)	1	34.86
6	*A*_*2*_ (5m)	*B*_*3*_ (100%)	*C*_*1*_ (0.5GPa)	2	30.96
7	*A*_*3*_ (7m)	*B*_*1*_ (60%)	*C*_*2*_ (1.0GPa)	2	40.34
8	*A*_*3*_ (7m)	*B*_*2*_ (80%)	*C*_*1*_ (0.5GPa)	3	36.64
9	*A*_*3*_ (7m)	*B*_*3*_ (100%)	*C*_*3*_ (1.5GPa)	1	31.44
*T*_*1*_ *T*_*2*_ *T*_*3*_	541.8	674.94	613.38	605.58	
	620.04	604.56	609.66	603.36	
	650.52	532.86	589.32	603.42	
*K*_*ij*_	90.3	112.49	102.23	100.93	
	103.34	100.76	101.61	100.56	
	108.42	88.81	98.22	100.57	
*R*	18.12	23.68	4.01	0.37	

Note: *T*_*i*_ represents the sum of indicators for each level of the main control factor, where *i* = 1,2,3; *K*_*ij*_ represents the mean value of the peak support stress at the j^th^ level of the i^th^ main control factor, where *i* = 1,2,3..., *j* = 1,2,3; *R* represents the range value of the experimental factors at various levels, calculated as *R*_e_* = max(K*_*i1,*_ *K*_*i2,*_ *K*_*i3*_*)- min(K*_*i1,*_ *K*_*i2,*_ *K*_*i3*_*),* where *i = *1, 2,3..., e = *A*,*B*,*C*...

A larger *R*_e_ indicates a greater impact of changes in the factor’s level on the depth of floor failure, indicating a higher degree of sensitivity. The results of the range analysis are presented in Table 4. The range value for the empty column is 0.37, substantially smaller than those of the other factors, suggesting that interactions among the factors can be disregarded. Specifically, the range values for mining height, backfill collapse ratio(*η*), and elastic modulus of the filling material are 18.12, 23.68, and 4.01 respectively. The order of range values for the main controlling factors, which reflects their sensitivity, is *R*_*B*_ > *R*_*A*_ > *R*_*C*_. This indicates that the backfill collapse ratio is the most sensitive factor, followed by mining height, and then the elastic modulus of the filling material.

According to the basic principles of mathematical statistics, the indicator level matrix *M*, the factor level matrix *T*, and the level level matrix *S* are established, and the weight matrix is noted as *Y*.


Y=MTS (25)

$M=\left[\begin{array}{ccccc} {}*20ck_{11} & 0 & 0 & \cdots & 0\\ \vdots & \vdots & \vdots & \cdots & \vdots \\ k_{1n} & 0 & 0 & \cdots & 0\\ 0 & k_{21} & 0 & \cdots & 0\\ \vdots & \vdots & \vdots & \cdots & \vdots \\ 0 & k_{2n} & 0 & \cdots & 0\\ \vdots & \vdots & \vdots & \ddots & \vdots \\ 0 & 0 & 0 & \cdots & k_{m1}\\ \cdots & \cdots & \cdots & \cdots & \cdots \\ 0 & 0 & 0 & \cdots & k_{mn} \end{array}\right]$ $T=\left[\begin{array}{cccc} {}*20cT_{1} & 0 & \cdots & 0\\ 0 & T_{2} & \cdots & 0\\ \vdots & \vdots & \ddots & \vdots \\ 0 & 0 & \cdots & T_{m} \end{array}\right]$ $S=\left[\begin{array}{c} {}*20cS_{1}\\ S_{1}\\ \vdots \\ S_{m} \end{array}\right]$

In the above matrix, m and n are the number of factors and levels in the orthogonal test, respectively, and in this orthogonal test, *m* = 3 and *n* = 3; the index matrix *M* examines support stress peak value, and the smaller the support stress peak value represents the more stable, so $k_{ij}=1/K_{ij}$; the factor matrix $T_{i}=1/\sum {\mathrm{\backslash nolimits}}_{j=1}^{n}1/K_{ij}$; the level matrix $S_{i}=R_{i}/\sum {\mathrm{\backslash nolimits}}_{i=1}^{n}R_{i}$.

Bringing the corresponding data from the master factor polar analysis table into equation (25) yields the subgrade damage depth influence weights:


Y=MTS=[0.267,0.251,0.236,0.344,0.357,0.368,0.0849,0.0871,0.0884]

The larger the weight value of the level in the main control factors, the smaller the influence of the level on the support stress peak value. According to the results of the weight calculation, the three factors with the largest weights are *A*_1_, *B*_3_ and *C*_3_, so the optimal scheme of the orthogonal simulation test can be determined to be *A*_1_*B*_3_*C*_3_, which means that when the mining height is 3m, the filling span ratio is 100%, and the elastic modulus of the gangue is 1.5GPa, the peak value of the supporting stress is minimized, which can effectively reduce the risk of rock burst..

### 4.3 Variance analysis of main controlling factors for pressure manifestation in gangue backfill mining

Variance analysis is a method that assesses the significance of differences in the overall means at various levels of factors by comparing the magnitude of data fluctuations caused by those factors to those resulting from errors. The variance analysis of main controlling factors in strata pressure behavior in backfill mining is shown in Table 6.

**Table 6:** Variance analysis of main controlling factors for pressure manifestation.

Experiment Group	Main Control Factor Levels				
	Mining Height/m	Backfill Collapse Ratio	Elastic Modulus of Filling Body/GPa	Empty Column	Support Stress Peak Value/MPa
1	*A*_*1*_ (3m)	*B*_*1*_ (60%)	*C*_*1*_ (0.5GPa)	1	34.63
2	*A*_*1*_ (3m)	*B*_*2*_ (80%)	*C*_*3*_ (1.5GPa)	2	29.26
3	*A*_*1*_ (3m)	*B*_*3*_ (100%)	*C*_*2*_ (1.0GPa)	3	26.41
4	*A*_*2*_ (5m)	*B*_*1*_ (60%)	*C*_*3*_ (1.5GPa)	3	37.52
5	*A*_*2*_ (5m)	*B*_*2*_ (80%)	*C*_*2*_ (1.0GPa)	1	34.86
6	*A*_*2*_ (5m)	*B*_*3*_ (100%)	*C*_*1*_ (0.5GPa)	2	30.96
7	*A*_*3*_ (7m)	*B*_*1*_ (60%)	*C*_*2*_ (1.0GPa)	2	40.34
8	*A*_*3*_ (7m)	*B*_*2*_ (80%)	*C*_*1*_ (0.5GPa)	3	36.64
9	*A*_*3*_ (7m)	*B*_*3*_ (100%)	*C*_*3*_ (1.5GPa)	1	31.44
*J*_*1*_ *J*_*2*_ *J*_*3*_	361.2	449.96	408.92		*Q* = 302.6
	413.36	403.04	406.44		*Q*^*2*^ = 91240.24
	433.68	355.24	392.88		*U* = 10137.80
*J*_*11*_	8154.09	12654.00	10450.97		
*J*_*22*_	10679.15	10152.58	10324.59		
*J*_*33*_	11754.90	7887.22	9647.17		
*S*_*I*_	58.24	90.98	3.10		
*S*_*R*_					154.84
*S*_*E*_					2.52

Notes: *J*_*i*_ represents the sum of the experimental indices for the damage depth of the bottom plate at the same level for each main controlling factor, where *i* = 1,2,3; *Q* represents the sum of the peak support stress values for the nine test schemes, $Q=\sum {\mathrm{\backslash nolimits}}_{k=1}^{9}y_{k}$;_._ *U* is the correction factor, calculated as *U = Q*^*2*^*/*9; *S*_*i*_ represents the sum of squares of deviations for each factor, $S_{R}=\sum {\mathrm{\backslash nolimits}}_{k=1}^{9}y_{k}^{2}-c$; *S*_*E*_ represents the sum of squares of deviations due to experimental error, calculated as *S*_*E=*_*S*_*R*_*-S*_*I.*_.

Significance testing can eliminate the first and second types of errors commonly found in orthogonal experiments. The calculation method is as follows:


$$F=\frac{S_{i}/F_{i}}{S_{e}/F_{e}}$$ (25)

Where *F* represents the ratio of the mean square of deviations for each factor to the mean square of deviations due to experimental error. $S_{i}$ and $F_{i}$ denote the sum of squares of deviations and degrees of freedom for each factor respectively, while *S*_e_ and *F*_e_ represent the sum of squares of deviations and degrees of freedom for experimental error. The critical values for the F-test are *F*_0.01_ (2,4)=18 and *F*_0.05_ (2,4)=6.94. If *F* >* F*_0.01_ (*f*_i_, *f*_e_), the factor is considered highly significant and is indicated by ‘**’; if *F* <* F*_0.05_ (*f*_i_, *f*_e_), the factor is not significant.

The *F*-values of the main controlling factors are shown in Table 7. The F-values for mining height, backfill collapse ratio, and coal gangue elastic modulus are 23.02, 36.94, and 1.23, respectively. The sensitivity ranking is backfill collapse ratio > mining height > coal gangue elastic modulus. Consistent with the results of the range analysis method.

**Table 7:** Significance test of main controlling factors for pressure manifestation.

main Controlling Factor	Sum of Squares	Degrees of Freedom	Mean Square	*F*	*F*_0.01_(2,4)	*F*_0.05_(2,4)	Significance
*A*	58.24	2	29.12	23.02	18	6.94	**
*B*	93.46	2	46.73	36.94			**
*C*	3.10	2	1.55	1.23			
Error e	2.52	2	1.26				

**Fig 8:**
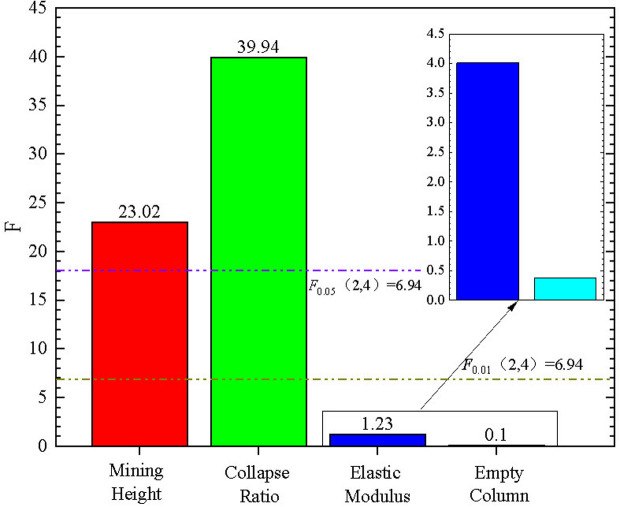
Significance analysis of the main control factors.

The significance analysis of the main control factors is shown (Fig 8). Among them, *F*_*A*_ and *F*_*B*_ are greater than *F*_0.01_(2,4), which means that the influence of mining height and filling ratio on the support stress peak value among the main control factors is highly significant, and *F*_*C*_ is less than *F*_0.05_(2,4), which indicates that the elastic modulus of the filling body does not have a significant influence on the peak bearing stress.

### 4.4 Sensitivity research analysis

Based on establishing an orthogonal experimental model, range and variance analyses were conducted on mining height, impact ratio, and elastic modulus of gangue as research objects. Both analysis methods obtained the sensitivity ranking of the main controlling factors as follows: filling ratio>mining height>filling elastic modulus. In addition, the variance analysis method showed that mining height and filling ratio had a highly significant impact on the peak support stress. For practical engineering projects with complexity and uncertainty, the research results suggest that, while ensuring economic benefits, in order to ensure the stability of coal seams under backfill mining, the first consideration should be to increase the filling span ratio, and finally to increase the elastic modulus of gangue.

## 5. Summary

In order to study the mechanism of rock pressure weakening and the sensitivity of main control factors, a mechanical model is constructed, and the calculation method of rock pressure under filling mining is derived. Then, the law of rock pressure weakening under filling mining was analyzed. Finally, the sensitivity of each main control factor to the manifestation of rock pressure under filling mining was obtained. The study can provide theoretical support for the stability control of coal seams under filling mining. The main conclusions are as follows:

(1) Under the support of coal gangue, the vertical stress in the coal seam exhibits an arched shape. When compared to conventional caving mining, the peak vertical stress in the coal seam after gangue filling is approximately 70.91% of that in non-filled conditions.
(2) Gangue filling can provide support stress to the overlying rock strata, effectively reducing the damage extent. The range of plastic damage is relatively stable, and the primary failure mode in the plastic zone of roof is shear failure.
(3) Range analysis shows that the sensitivity ranking is backfill collapse ratio > mining height > filling body elastic modulus. Therefore, in practical engineering, in order to ensure the stability of coal seams under backfill mining while ensuring economic benefits, the first consideration should be to increase the filling span ratio, and finally to increase the elastic modulus of gangue.
(4) This study is mainly based on a combination of theoretical calculations and numerical simulations, lacking sufficient engineering examples for verification. Subsequently, relevant scholars can use this research method for comparative analysis based on actual working conditions.

## Supporting information

S1 File Significance analysis data. (XLSX)

S2 File Range analysis data. (XLS)

S3 File Peak stress of orthogonal experimental group. (XLS)

S4 File FLAC3D code. (TXT)

S5 File Analysis data. (XLSX)
