# Correction: Study on the mechanism of mining-induced pressure mitigation in gangue backfill mining and the sensitivity analysis of main controlling factors

**DOI:** 10.1371/journal.pone.0343342

**Published:** 2026-02-17

**Authors:** Lei Zhu, Yunong Xu, Chengyong Liu, Yuejin Zhou, Wenzhe Gu, Cunli Zhu, Zhicheng Liu

There is an error in affiliation 1 for authors Lei Zhu, Chengyong Liu, Wenzhe Gu and Zhicheng Liu. The correct affiliation 1 is: China Coal Energy Research Institute Co., Ltd, Xi’an, China.
